# Why We Must Talk About Institutional Corruption to Understand Wrongdoing in the Health Sector: Comment on "We Need to Talk About Corruption in Health Systems"

**DOI:** 10.15171/ijhpm.2019.103

**Published:** 2019-11-16

**Authors:** Marianna Fotaki

**Affiliations:** Warwick Business School, University of Warwick, Coventry, UK.

**Keywords:** Institutional Corruption, Policy, Legislation, Healthcare, Wrongdoing, Illegal

## Abstract

While various forms of corruption are common in many health systems around the world, defining wrongdoing in terms of legality and the use of public office for private gain obstructs our understanding of its nature and intractability. To address this, I suggest, we must not only break the silence about the extent of wrongdoing in the health sector, but also talk differently about corruption in general, and corruption in healthcare specifically. I propose adopting the notion of institutional corruption (IC) developed by Thompson and Lessig, as divergence from the original purpose of the institution, which may not be illegal but may nevertheless cause harm to people who depend on it by creating perverse dependencies and compelling individuals to act against its core purpose. Such work is much needed to provide in-depth accounts of how external political and legislative pressures enable corruption in healthcare systems. I also argue for bringing together insights from various research domains and levels of analysis to capture why and how corruption becomes systemic, deeply embedded, and intractable.

## What Is Wrong With Corruption?

Corruption evokes strong feelings and undesirable associations. The word itself conjures something powerful, insidious, and destructive of human lives and institutions.^1^ In addition to the factors identified by the authors in their recent editorial,^2^ this perhaps explains the secrecy that surrounds it. Corruption is antithetical to the notion of healing,^3^ causing harm to those in need of care and diminishing trust in the health system,^4^ which thereby deviates from its core purpose of improving lives. Although its negative impact on society and individual welfare is well-established,^5,6^ corruption is a pervasive problem in the health sector.^7^ To address this, I suggest, we must not only break the silence about the extent of wrongdoing in the health sector, but also talk differently about corruption in general, and corruption in healthcare specifically.

Corruption commonly refers to illegal activities taking various forms – petty or grand, covert or open, limited or extensive, black, grey or white, individual or systemic.^8,9^ It may be acceptable, harmful, or simply routine.^10^ Transparency International’s widely adopted definition^11^ is “the abuse of entrusted power for private gain.” However, this definition fails to acknowledge that corruption not only or even mainly happens in the public sector, nor does it concern the non-Western other^3,12^; The consequences of these for the public are probably far greater than in instances of individual corruption and wrongdoing, even if such actions are not unlawful, since they may lead, for example, to the public funding less cost-effective care.

Arguably, corruption is not antithetical to advanced capitalism, involving prestigious institutions and stalwart private enterprises in many developed countries. Thus, although developed democracies may have reduced the incidence of conventional corruption, they are prone to their own kind of corruption, which may be more insidious.^13^ These insidious forms of corruption are usually socially sanctioned and perfectly legal. The theory of institutional corruption (IC)^14^ stresses the impact of policy incentives and regulation on organizational culture, and how they may cause organizations to diverge from their original purpose. It can be usefully applied to better understand how organizations tasked with protecting the public interest in the health sector may lose credibility if they depart from their original mission by engaging in activities that endanger it, even if these activities are not illegal.

For instance, in the United States, members of the Food and Drug Administration’s advisory committees on drug approval are known to have financial ties with pharmaceutical companies.^15^ According to an analysis of committee records from 2000, in 55% of meetings, half or more of the advisors had conflicts of interest.^16^ Although their actions may not be considered corrupt in the conventional sense, these connections have been shown to influence government decision-making.^15,17^ There is evidence that corruption can be found within any enabling system, including the behavior of political and administrative actors as purchasers, and various public, private, and non-profit organizations as providers of public services.^18^

This suggests that conceiving corruption only in the context of illegal activities and obtaining private gain is too narrow. It does not include policy and legislative influences that undermine organizations and institutions tasked with protecting the public interest, leading to systematic divergence from their role as they engage in activities that are not illegal yet jeopardize their stated tasks. Deviation from organizational purpose with or without involving direct personal gain, may pervert the moral fabric of institutions and organizations, raising questions about their raison d’être; and such environments may create yet more fertile conditions for organizational wrongdoing by unscrupulous individuals. For these reasons, I call for the notion of corruption in the health sector to be extended beyond “illegal” and unethical forms of wrongdoing, to reconsider how forces both within and outside health institutions create situations that allow or encourage organizations and individuals to deviate from their core purpose.

## Why Institutional Corruption Matters?

Many disciplines have attempted to explain corruption. One broad strand is concerned with structural regulatory failure, poorly designed incentives, and the politics of corruption.^4,19^ The broader literature on wrongdoing in organizations focuses largely on the question of why individuals engage in wrongdoing.^20,21^ While providing important insights, these theories leave gaps in understanding of the organizational conditions that encourage corrupt practices to flourish. There remains the question of how collective wrongdoing spreads across individuals, such that they come to work together to do wrong in the name of the organization.^22^

The theory of IC provides an answer to this,^13,14,23^ focusing on forms of corruption that are not strictly illegal yet pervert an institution’s function under conditions that may promote personal benefit. It was originally developed to theorize the nature of corruption in the US Congress, explaining why organizational members are often trapped in finance-related institutional dependencies, for example relying on money from major donors or special interest groups for election and/or retaining office.^23^ Unlike bribery, campaign fundraising serves a legitimate function and is perfectly legal, since political institutions rely on campaign donations for their functioning. Yet the influence of money damages democracy by circumventing and/or bypassing the legislative process and breaking the link between representation and power. According to legal scholar, Lawrence Lessig, who extended its application to a wider range of institutions, this form of corruption:

*“…is a systemic and strategic influence which is legal, or even currently ethical, that undermines the institution’s effectiveness by diverting it from its purpose or weakening its ability to achieve its purpose, including, to the extent relevant to its purpose, weakening either the public’s trust in that institution or the institution’s inherent trustworthiness.”* ^23^

Institutional members have considerable discretion over the extent of their involvement in IC, ranging from extracting maximum benefit from the existing system, to minimal compliance, or speaking out and engaging in various other activities to counteract it.^24^ Organizational leaders may purposely manipulate administrative structures to control corruption.^25^ Individuals may also become involved in corrupt behaviors on behalf of the institution,^26^ as they engage in self-deception to serve their own best interests.

The theory of IC, emphasizing the potential impact of an “economy of influence” that undermines institutions’ ability to perform their core tasks, has been extended to explain wrongdoing in many other public- and private-sector fields, including health services and the pharmaceutical industry.^15,16^ It offers unique insights into the causes and dynamics of corruption in health systems by enriching existing research. It elucidates how organizations themselves interact with regulatory regimes, public policies, and wider societal discourses through their leaders and members in ways that contribute to institutions’ divergence from their legitimate purpose. Such practices are a source of organizational corruption, because they provide benefits that even an uncorrupted institution needs, thus creating perverse dependencies and compelling individuals to act against the organizational purpose.^23^ In addition to undermining their purpose, such forms of corruption often harm the very people who rely on them. This is important, because the failure of designated institutions to protect the public interest undermines the principles of good governance^19^ and perverts the rule of law.^27^ Its corrosive effects on public trust and the ethical conduct of individuals in organizations may lead to the breakdown of the entire system if a perception of endemic corruption prevails.^28^ Overall, this form of corruption benefits the institution, while undermining it by putting its long-term survival at risk.^29^

To counteract such undesirable developments and redress the damage caused by loss of public confidence in in health organizations and health systems,^30^ the dynamics and mechanisms that cause them to become corrupt must be fully understood. Given the complexity and the multifaceted and multidimensional nature of corruption, we must develop transdisciplinary frameworks to address this. For instance, drawing on political economy and legal studies is important for understanding macro influences from policy and legislation; while institutional theory and organization studies can explain their implementation; and behavioural ethics and anthropology shed light on individual motivations in different cultures and sectors. Unless insights from various research domains and levels of analysis are brought together, we may not capture why and how corruption becomes systemic, deeply embedded, and intractable.^28^

Adopting a definition of IC as deviation from organizational purpose with or without involving direct personal gain^23,24^ to theorize wrongdoing in public health institutions would allow us to capture the processes, factors, and institutional conditions that may foster corruption, irrespective of their legality or ethicality. This may also help us identifing organizational structures, modes of operation, and initiatives that promote ethical behavior and remedy corruption when it has taken root. Drawing on various disciplinary insights and findings from across sectors will help model the consecutive steps required to introduce specific measures. The practical outcome of such an approach would be to design tools and methodologies to prevent and/or remedy IC, which may be of use to policy-makers and organizations.

In conclusion, I would like reiterate the necessity to examine “legal” forms of corruption in public institutions which causes them to systematically deviate from their core purpose, damaging their trustworthiness. I also argue for closer integration of the IC frame with organizational and ethical perspectives on wrongdoing in health systems, to better understand how policy influences managerial decisions and individual behaviors in healthcare organizations. Such integration might help prevent unethical outcomes and damage to society by restoring public institutions to their primary purpose (eg, delivering high-quality care in a cost-effective manner), rather than making them less able to perform their functions. Finally, this might form a basis for developing of a set of practice-orientated diagnostic tools and comprehensive interventions, aiming to prevent and address IC where it occurs.

## Ethical issues

Not applicable.

## Competing interests

Author declares that she has no competing interests.

## Author’s contribution

MF is the single author of the paper.

## References

[1] Underkuffler LS. Captured by evil: The idea of corruption in law. Duke Law School Faculty Scholarship Series 2005; 23. https://lsr.nellco.org/duke_fs/23.

[2] Hutchinson E, Balabanova D, McKee M (2019). We need to talk about corruption in health systems. Int J Health Policy Manag.

[3] Chattopadhyay S (2013). Corruption in healthcare and medicine: Why should physicians and bioethicists care and what should they do?. Ind J Med Ethics.

[4] Rose-Ackerman S. Corruption and Government: Causes, Consequences, and Reform. Cambridge, UK: Cambridge University Press; 1999.

[5] Rose-Ackerman S, Palifka BJ. Corruption and Government: Causes, Consequences, and Reform. 2nd ed. New York, NY: Cambridge University Press; 2016.

[6] Transparency International. Diagnosing Corruption in Healthcare. London: Transparency International UK; 2016.

[7] Vian T (2008). Review of corruption in the health sector: Theory, methods and interventions. Health Policy Plan.

[8] Tanzi V, Davoodi H. Corruption, public investment and growth. IMF Working Paper 97/139. Washington, DC: International Monetary Fund; 1997.

[9] Campos JE, Pradahan P. The Many Faces of Corruption: Tracking Vulnerabilities at the Sector Level. Washington, DC: World Bank; 2007.

[10] Graycar A, Prenzler T. Understanding and Preventing Corruption. London: Palgrave; 2013.

[11] Transparency International. The Anti-Corruption Plain Language Guide. Berlin, Germany: Transparency International; 2009.

[12] Haller D, Shore C, eds. Corruption: Anthropological Perspectives. London: Pluto Press; 2005.

[13] Thompson D (2019). Theories of institutional corruption. Annu Rev Polit Sci.

[14] Thompson D. Ethics in Congress: From Individual to Institutional Corruption. Washington, DC: Brookings Institution; 1995.

[15] Angell M. The Truth About the Drug Companies: How They Deceive Us and What to Do About It. New York, NY: Random House; 2004.

[16] Rodwin MA (2012). Conflicts of interest, institutional corruption, and pharma: An agenda for reform. J Law Med Ethics.

[17] Kassirer J. The corrupting influence of money in medicine. In: Transparency International, ed. Global Corruption Report 2006, Special Focus on Corruption and Health. London: Pluto Press; 2006.

[18] Von Maravic P, Reichard C (2003). New Public Management and corruption: International Public Management Network (IPMN) dialogue and analysis. Int Public Manag Rev.

[19] Della Porta D, Mény Y, eds. Democracy and Corruption in Europe. London: Cassell; 1997.

[20] Fleming P, Zyglidopoulos S (2009). Rationalization, overcompensation and the escalation of corruption in organizations. J Bus Ethics.

[21] Warren DE (2003). Constructive and destructive deviance in organizations. Acad Manag Rev.

[22] Smith-Crowe K, Warren DE (2014). The emotion-evoked collective corruption model: The role of emotion in the spread of corruption within organizations. Organ Sci.

[23] Lessig L. Republic, Lost: How Money Corrupts Congress – And a Plan to Stop It. New York, NY: Twelve Books; 2011.

[24] Light DW. Strengthening the theory of institutional corruptions: Broadening, clarifying, and measuring. Working Paper No. 2, Edmond J Safra Center for Ethics. Boston, MA: Harvard University; 2013.

[25] Lange D (2008). A multidimensional conceptualization of organizational corruption control. Acad Manag Rev.

[26] Pinto J, Leanna CR, Pil KF (2008). Corrupt organizations or organizations of corrupt individuals? Two types of organization-level corruption. Acad Manag Rev.

[27] United Nations Development Programme. Institutional Arrangements to Combat Corruption: A Comparative Study. Bangkok, Thailand: UNDP Regional Center in Bangkok; 2005.

[28] Ashforth B, Gioia DA, Robinson SL, Trevino L (2008). Re-viewing institutional corruption. Acad Manag Rev.

[29] Fotaki M. Counteracting institutional corruption in diverse organizational and cultural settings. Project Report, Harvard University: Edmond J. Safra Center for Ethics; 2015.

[30] Fotaki Fotaki, M M (2014). Can consumer choice replace trust in the National Health Service in England? Towards developing an affective psychosocial conception of trust in health care. Sociol Health Illn.

